# Low triiodothyronine syndrome is associated with hemorrhagic transformation in patients with acute ischaemic stroke

**DOI:** 10.18632/aging.102195

**Published:** 2019-08-27

**Authors:** Gui-Qian Huang, Ya-Ying Zeng, Qian-Qian Cheng, Hao-Ran Cheng, Yi-Ting Ruan, Cheng-Xiang Yuan, Yun-Bin Chen, Wei-Lei He, Hui-Jun Chen, Jin-Cai He

**Affiliations:** 1 Department of Neurology, The First Affiliated Hospital of Wenzhou Medical University, Wenzhou 325000, China; 2 School of Mental Health, Wenzhou Medical University, Wenzhou 325000, China

**Keywords:** low triiodothyronine syndrome, hemorrhagic transformation, acute ischemic stroke, thyroid hormone

## Abstract

Hemorrhagic transformation (HT) is a severe complication occurring in acute ischemic stroke (AIS) patients. We explored the association between low triiodothyronine (T3) syndrome and HT in AIS patients. A total of 208 consecutive participants with HT and 208 age- and sex-matched stroke patients without HT were enrolled in this study. HT was diagnosed by follow-up imaging assessment, and was radiologically classified as hemorrhagic infarction (HI) type 1 or 2 or parenchymal hematoma (PH) type 1 or 2. HT was also classified into asymptomatic or symptomatic. The incidence of low T3 syndrome was significantly higher among patients who developed HT than among those without HT. Moreover, the more severe the HT, the lower the detected T3 levels. Multivariate-adjusted binary logistic regression showed that low T3 syndrome was an independent risk factor for HT and symptomatic HT in AIS patients. Low T3 syndrome was also significantly associated with a higher risk of PH, but not with the risk of HI. Thus, low T3 syndrome was independently associated with the risk of HT, symptomatic HT, and severe HT (PH) in AIS patients, which suggests monitoring T3 could be a useful means of preventing HT in patients with ischemic stroke.

## INTRODUCTION

Hemorrhagic transformation (HT) is a common complication among patients suffering from acute ischemic stroke (AIS) [1, 2], and has been shown to result in poor outcomes, including early mortality and disability [3–5]. Therefore, to help clinicians predict prognosis and provide appropriate treatment and to reduce the incidence of HT in AIS patients, it is necessary that we identify the risk factors for HT.

Risk factors for HT previously identified in ischemic stroke patients include old age [6], atrial fibrillation [4], prolonged interval between stroke onset to treatment [5], elevated blood glucose [7], higher systolic blood pressure [8], thrombolysis [9], and symptom severity [10]. A recent study of 20 patients with spontaneous subarachnoid hemorrhage suggested that low serum triiodothyronine (T3) concentrations were associated to more serious subarachnoid hemorrhage and poor clinical outcomes [11]. Another study reported that after adjustment for potential confounding factors, low free T3 was identified as an independent risk factor for symptomatic intracranial hemorrhage [12]. In fact, some studies have found that thyroid hormone deficiency may affect vascular endothelial function [13, 14]. Given that disruption of vascular endothelial cells is a crucial mechanism related to the development of HT after cerebral ischemia [15], the presence of low thyroid hormone levels may indicate an increased risk of HT.

Low T3 syndrome, also known as euthyroid sick syndrome or non-thyroidal illness syndrome, is characterized by low levels of T3 with normal concentrations of thyroid stimulating hormone (TSH) [16], which is a typical pattern of thyroid hormone abnormalities seen in patients with critical disease [17]. In addition, it has been widely reported that low T3 syndrome occurs in patients with cerebrovascular illnesses, including stroke [18–20].

In the present study, therefore, we hypothesized that low T3 syndrome may be associated with HT in patients with AIS. To address that issue, we investigated the effect of low T3 syndrome on HT in a population of AIS patients.

## RESULTS

### Classification of patients with HT

In this study, 312 (72.6%) subjects were male and 104 (27.4%) were female. The mean ages of patients with and without HT were 68.7 and 68.6 years, respectively. Among the patients with HT, 83 (39.9%) were classified as having symptomatic HT (sHT), while 125 (60.1%) had asymptomatic HT (asHT). Based on the imaging features, hemorrhagic infarct type 1 (HI-1) occurred in 50 (24.0%) patients, hemorrhagic infarct type 2 (HI-2) in 64 (30.8%), parenchymal hematoma type 1 (PH-1) in 48 (23.1%), and parenchymal hematoma type 2 (PH-2) in 46 (22.1%).

### Baseline characteristics

The demographic, clinical, and laboratory characteristics of the patients with and without HT are summarized in Table 1. Compared to the patients without HT, those with HT were more likely to undergo atrial fibrillation or anticoagulation therapy, and were less likely to undergo antiplatelet or lipid-lowering therapies. They also had higher baseline leukocyte counts; higher fibrinogen, total cholesterol, and glucose levels; and higher initial National Institutes of Health Stroke Scale (NIHSS) scores. In addition, fewer cigarette smokers and alcohol drinkers were identified among patients with HT.

**Table 1:** Differences of the baseline characteristics in AIS patients with and without HT.

**Variables**	**Non-HT (n = 208)**	**HT (n = 208)**	P-value*
**Demographic parameters**			
Age (years)	68.6 ± 11.5	68.7 ± 11.6	0.922
Male, n (%)	151 (72.6%)	151 (72.6%)	1.000
BMI (kg/m^2^)	23.6 ± 3.2	23.0 ± 3.2	0.166
Marital status			0.283
Married, n (%)	194 (93.3%)	199 (95.7%)	
**Vascular risk factors**			
History of atrial fibrillation, n (%)	19 (9.2%)	73 (35.1%)	<0.001
History of hypertension, n (%)	146 (70.2%)	140 (67.3%)	0.526
History of diabetes, n (%)	53 (25.5%)	58 (27.9%)	0.579
History of dyslipidemia, n (%)	12 (5.8%)	16 (7.7%)	0.426
Current smoking, n (%)	120 (58.0%)	97 (47.3%)	0.030
Current drinking, n (%)	119 (61.3%)	84 (41.0%)	<0.001
**Biochemistry and vital signs on admission**			
Baseline SBP (mmHg)	147.4 ± 22.4	149.6 ± 22.0	0.318
Baseline DBP (mmHg)	82.2 ± 14.0	83.3 ± 13.9	0.398
Leukocyte counts (×109/L)	6.7 ± 1.8	8.6 ± 3.2	<0.001
Platelets (×109/L)	209.0 ± 54.5	201.5 ± 65.7	0.205
Hgb (g/L)	136.3 ± 14.6	137.4 ± 15.4	0.457
Fibrinogen (g/L)	3.4 ± 0.9	4.1 ± 1.4	<0.001
Glucose levels (mmol/L)	5.5 ± 1.6	6.6 ± 2.8	<0.001
Total cholesterol (mmol/L)	2.2 ± 1.6	3.7 ± 1.7	<0.001
TSH (mIU/L)	1.7 ± 1.1	1.7 ± 2.3	0.970
T4 (nmol/L)	105.7 ± 21.1	108.5 ± 21.4	0.193
T3 (nmol/L)	1.4 ± 0.3	1.1 ± 0.3	<0.001
Free T4 (pmol//L)	11.1 ± 1.8	12.2 ± 2.9	<0.001
Free T3 (pmol/L)	4.3 ± 0.6	4.1 ± 0.8	0.006
Low T3 syndrome, n (%)	68 (32.7%)	139 (66.8%)	<0.001
NIHSS on admission, median (IQR)	4.0 (1.0–8.0)	5.0 (2.0–10.0)	0.012
**Stroke mechanisms**			0.107
Atherosclerotic, n (%)	171 (82.2%)	158 (76.0%)	
Cardioembolic, n (%)	29 (13.9%)	46 (22.1%)	
Lacunar, n (%)	3 (1.4%)	1 (0.5%)	
Other causes, n (%)	5 (2.5%)	3 (1.4%)	
**Initial treatment in hospital**			
Antiplatelets, n (%)	189 (90.9%)	115 (55.3%)	<0.001
Anticoagulants, n (%)	18 (8.7%)	53 (25.5%)	<0.001
Lipid-lowering agents, n (%)	201 (96.6%)	183 (88.0%)	<0.001

NOTE. BMI, body mass index; DBP, diastolic blood pressure; Hgb, hemoglobin; HT: hemorrhagic transformation; NIHSS, National Institutes of Health Stroke Scale; SBP, systolic blood pressure; T3, triiodothyronine; T4, total thyroxine; TSH: thyroid stimulating hormone. *Continuous variables were compared between the groups by the Student’s t-test or the Mann–Whitney test. The chi-square test was used for categorical variables.

### Association between low T3 syndrome and HT

**Figure 1:**
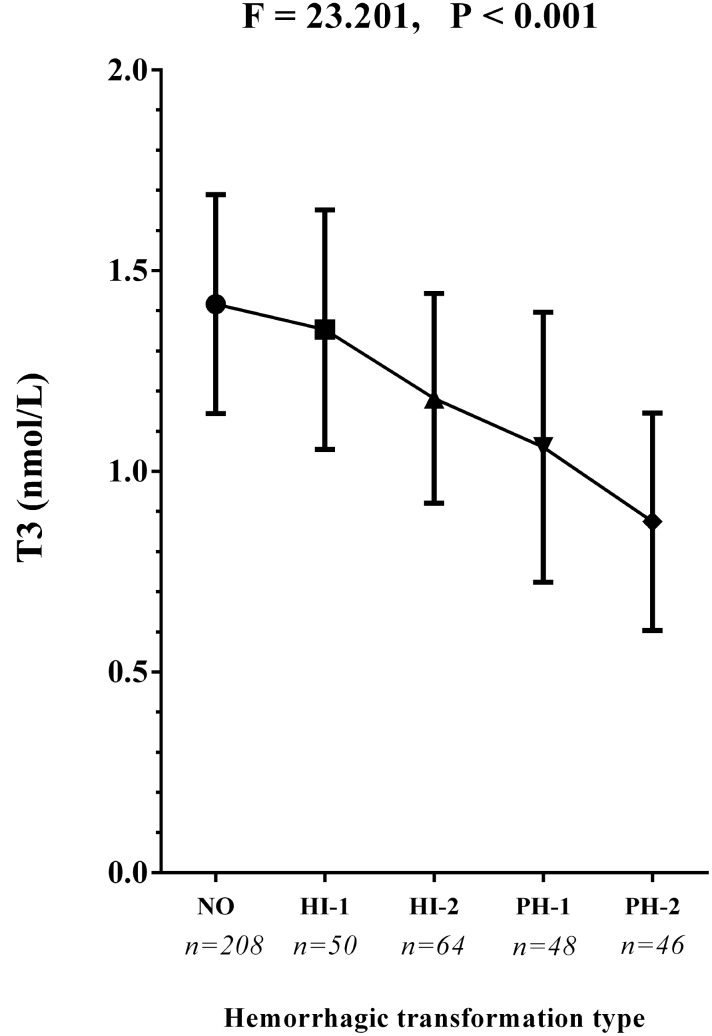
**The T3 concentrations in the subcategorized groups of HT.** Each data point and error bar correspond to the mean and standard deviation of T3 concentration by the subcategorized groups of HT. The line chart shows a gradual decease of T3 concentrations as the HT became more severe. HI-1, hemorrhagic infarct type 1; HI-2, hemorrhagic infarct type 2; PH-1, parenchymal hematoma type 1; PH-2, parenchymal hematoma type 2.

Baseline levels of total T3 and free T3 were significantly lower in patients with HT than without HT (1.1 mmol/l vs.1.4 mmol/l, P < 0.001; 4.1 mmol/l vs. 4.3 mmol/l, P < 0.001, respectively; Table 1). More patients with HT exhibited low T3 syndrome after ischemic stroke on admission than did those without HT (66.8% vs. 32.7%; chi-square test P <0.001; Table 1). As for the radiological status of HT, baseline levels of total and free T3 were also much lower in the subjects with PH than in those with HI (both P < 0.001; Table 2). Additionally, when compared to patients with HI, low T3 syndrome was more frequently detected in those with PH (P = 0.034; Table 2). Similar results were obtained upon comparison between patients with sHT and asHT . Figure 1 shows that T3 levels decreased in patients with HT as the condition worsened (P < 0.001 by ANOVA test).

**Table 2:** Comparison of the baseline characteristics according to the subcategorized groups of HT.

**Variables**	**Radiological HT**				**Clinical category**		
	**HI N = 114**	**PH N = 94**	P-value*		**asHT N = 125**	**sHT N = 83**	P-value*
**Demographic parameters**							
Age (years)	67.9 ± 11.5	69.7 ± 11.7	0.286		68.7 ± 13.0	68.7 ± 9.3	0.999
Male, n (%)	80 (70.2%)	71 (75.5%)	0.389		91 (72.8%)	60 (72.3%)	0.936
BMI (kg/m^2^)	23.1 ± 2.7	22.9 ± 3.9	0.868		23.2 ± 3.0	22.8 ± 3.5	0.561
Marital status							
Married, n (%)	111 (97.4%)	88 (93.6%)	0.305		119 (95.2%)	80 (96.4%)	0.681
**Vascular risk factors**							
History of atrial fibrillation, n (%)	32 (28.1%)	41 (43.6%)	0.019		35 (28.0%)	38 (45.8%)	0.009
History of hypertension, n (%)	80 (70.2%)	60 (63.8%)	0.332		81 (64.8%)	59 (71.1%)	0.344
History of diabetes, n (%)	30 (26.3%)	28 (29.8%)	0.578		35 (28.0%)	23 (27.7%)	0.964
History of dyslipidemia, n (%)	11 (9.6%)	5 (5.4%)	0.252		12 (9.7%)	4 (4.8%)	0.200
Current smoking, n (%)	52 (46.0%)	45 (48.9%)	0.680		62 (50.4%)	46 (56.1%)	0.424
Current drinking, n (%)	43 (38.1%)	41 (44.6%)	0.346		75 (61.0%)	46 (56.1%)	0.487
**Biochemistry and vital signs on admission**							
Baseline SBP (mmHg)	151.3 ± 21.4	147.4 ± 22.7	0.200		149.2 ± 22.0	150.1 ± 22.1	0.771
Baseline DBP (mmHg)	84.0 ± 12.5	82.5 ± 15.5	0.456		82.2 ± 14.1	84.9 ± 13.7	0.172
Leukocyte counts (×109/L)	8.3 ± 3.3	9.0 ± 3.1	0.118		8.2 ± 2.5	9.2 ± 4.0	0.022
Platelets (×109/L)	212.9 ± 64.5	187.6 ± 64.7	0.005		204.1 ± 66.9	197.5 ± 64.1	0.484
Hgb (g/L)	137.4 ± 14.4	137.5 ± 16.7	0.953		135.7 ± 15.3	140.0 ± 15.3	0.044
Fibrinogen (g/L)	4.1 ± 1.3	4.1 ± 1.5	0.973		4.1 ± 1.4	4.0 ± 1.3	0.697
Glucose levels (mmol/L)	6.2 ± 2.4	6.9 ± 3.2	0.065		6.4 ± 2.7	6.8 ± 2.9	0.326
Total cholesterol (mmol/L)	3.9 ± 1.6	3.5 ± 1.8	0.080		3.7 ± 1.7	3.8 ± 1.8	0.656
TSH (mIU/L)	1.9 ± 2.9	1.4 ± 0.9	0.079		1.7 ± 2.8	1.6 ± 1.1	0.747
T4 (nmol/L)	110.5 ± 22.7	106.1 ± 19.5	0.144		111.6 ± 22.2	103.6 ± 19.2	0.008
T3 (nmol/L)	1.3 ± 0.3	1.0 ± 0.3	<0.001		1.2 ± 0.4	1.0 ± 0.2	<0.001
Free T4 (pmol//L)	12.4 ± 3.3	12.0 ± 2.2	0.393		12.2 ± 3.3	12.1 ± 2.1	0.798
Free T3 (pmol/L)	4.3 ± 0.7	3.8 ± 0.7	<0.001		4.2 ± 0.8	3.9 ± 0.6	0.005
Low T3 syndrome, n (%)	69 (60.5%)	70 (74.5%)	0.034		72 (57.6%)	67 (80.7%)	<0.001
NIHSS on admission, median (IQR)	4.5 (2.0–9.8)	6.0 (3.0–11.0)	0.114		4.0 (2.0–11.0)	6.0 (3.0–9.5)	0.882
**Stroke mechanisms**			0.471				0.538
Atherosclerotic, n (%)	90 (78.9%)	68 (72.3%)			98 (78.4%)	60 (72.3%)	
Cardioembolic, n (%)	22 (19.3%)	24 (25.5%)			24 (19.2%)	22 (26.5%)	
Lacunar, n (%)	1 (0.9%)	0			1 (0.8%)	0	
Other causes, n (%)	1 (0.9%)	2 (2.2%)			2 (1.6%)	1 (1.2%)	
**Initial treatment before HT**							
Antiplatelets, n (%)	64 (56.1%)	51 (54.3%)	0.786		72 (57.6%)	43 (51.8%)	0.411
Anticoagulants, n (%)	24 (21.1%)	29 (30.9%)	0.107		31 (24.8%)	22 (26.5%)	0.782
Lipid-lowering agents, n (%)	102 (89.5%)	81 (86.2%)	0.466		111 (88.8%)	72 (86.7%)	0.656

NOTE. asHT, asymptomatic hemorrhagic transformation; BMI, body mass index; DBP, diastolic blood pressure; Hgb, hemoglobin; HI, hemorrhagic infarct; HT: hemorrhagic transformation; NIHSS, National Institutes of Health Stroke Scale; PH: parenchymal hematoma; SBP, systolic blood pressure; sHT: symptomatic hemorrhagic transformation; T3, triiodothyronine; T4, total thyroxine; T-C, total cholesterol; TSH: thyroid stimulating hormone. *Continuous variables were compared between the groups by the Student’s t-test or the Mann–Whitney test. The chi-square test was used for categorical variables.

Among the patients with low T3 syndrome, 68 (32.7%) did not have HT and were analyzed further. In the Supplementary Table 1, the baseline characteristics of AIS patients exhibiting low T3 syndrome with and without HT are compared. The results showed that the T3 levels in patients without HT were significantly higher than in those with HT (1.1 ± 0.1 nmol/L vs. 1.0 ± 0.2 nmol/L; P < 0.001). In addition, compared to patients with HT, those without HT were less likely to have atrial fibrillation, and they presented lower leukocyte counts; lower hemoglobin, glucose, T4, and total cholesterol levels; and lower initial NIHSS scores. Thus, the results from this subgroup were largely similar to those from the patient population as a whole (Table 1), with several exceptions, including current drinking, hemoglobin, total cholesterol, T4 and free T4.

**Figure 2:**
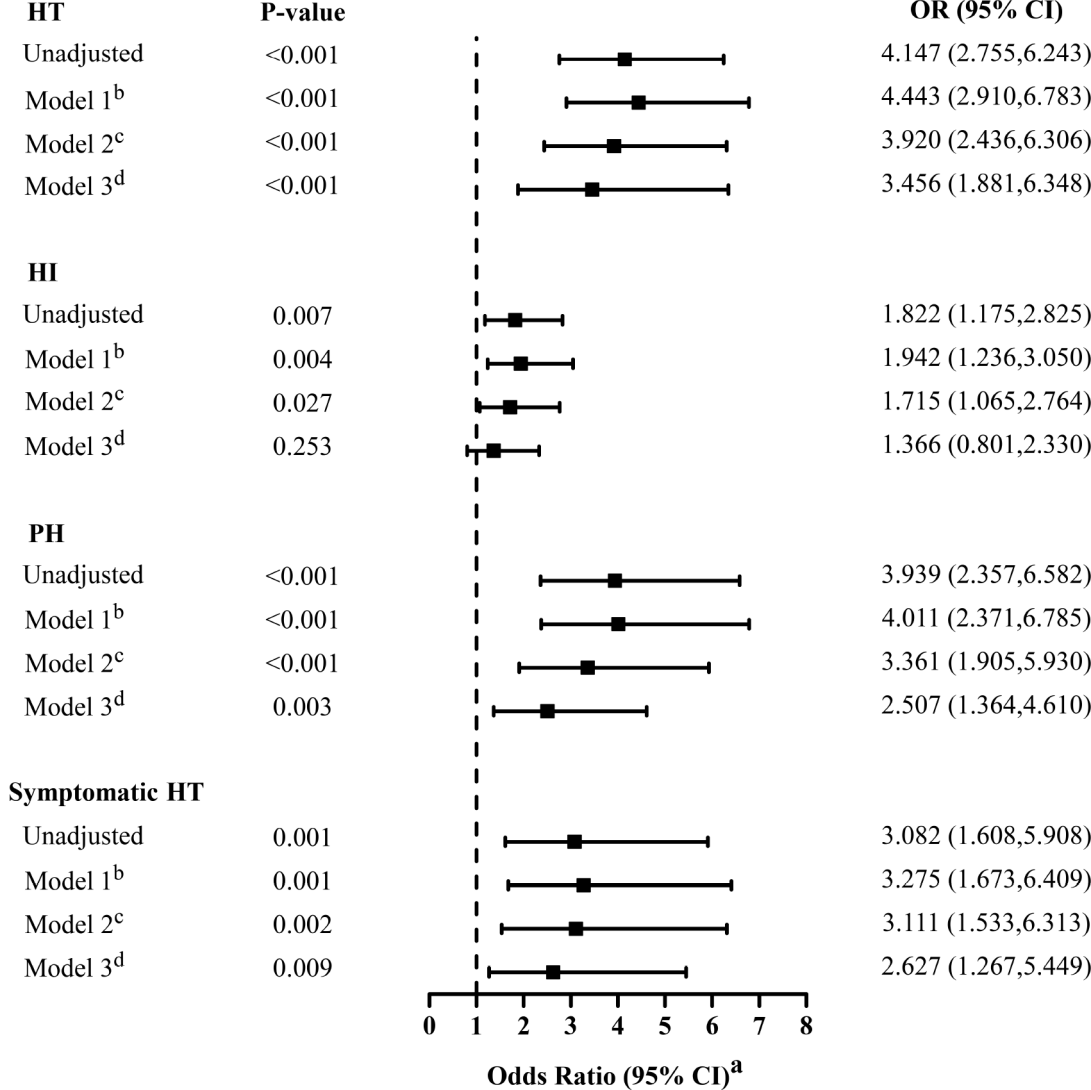
**Multivariate adjusted odds ratios for the association between low T3 syndrome and the subcategorized groups of HT (including HT, HI, PH and symptomatic HT respectively).** OR, odds radio; CI, confidence level; HI-1, hemorrhagic infarct type 1; HI-2, hemorrhagic infarct type 2; HT: hemorrhagic transformation; PH-1, parenchymal hematoma type 1; PH-2, parenchymal hematoma type 2. ^a^Reference OR (1.000) is the absence of low T3 syndrome for outcome. ^b^Model 1: adjusted for age, sex. ^c^Model 2: adjusted for covariates from Model 1 and further adjusted for identified risk factors for HT (systolic blood pressure, baseline NIHSS score, atrial fibrillation, Current smoking and current alcohol drinking). ^d^Model 3: adjusted for covariates from Model 2 and further adjusted for baseline leukocyte counts, fibrinogen, total cholesterol, the therapy of anticoagulant, antiplatelet and lipid-lowering.

In univariate analyses, low T3 syndrome at baseline was always significantly associated with radiological and clinical HT (OR = 4.147, 95% CI = 2.755–6.243, P < 0.001; OR = 3.082, 95% CI = 1.608–5.908, P = 0.001, respectively; Figure 2), and with a higher risk of PH (OR = 3.939, 95% CI = 2.357–6.582, P < 0.001; Figure 2) than HI (OR = 1.822, 95% CI = 1.175–2.825, P = 0.007; Figure 2). Atrial fibrillation, baseline NIHSS score, systolic blood pressure (SBP), smoking and drinking status, baseline leukocyte counts, fibrinogen, total cholesterol, and use of anticoagulant, antiplatelet or lipid-lowering agents were also significantly associated with HT.

A distinction was made between the three adjusted multivariable models, and for each dependent variable, the covariates for each model were the same (Figure 2). After initial adjustment for age and gender, it was found that the occurrence of low T3 syndrome was independently associated with HT (Model 1: OR = 4.443; 95% CI = 2.910–6.783, P < 0.001). The OR of HT for low T3 syndrome did not substantially change after further adjustment for factors already identified as risk factors for HT (Model 2: adjusting for age, gender, SBP, baseline NIHSS score, atrial fibrillation, smoke and drink status; OR = 3.920; 95% CI, 2.436–6.306; P < 0.001). In addition, the association was also apparent after adjusting for additional factors that significantly differed between patients with and without HT in the univariate analysis (Model 3: adjusting for covariates from Model 2 plus baseline leukocyte counts, fibrinogen, total cholesterol, anticoagulant, antiplatelet and lipid-lowering therapy; OR = 3.456, 95% CI = 1.881–6.348; P < 0.001). This result is similar to that obtained for patients with low T3 syndrome and sHT (Model 1: OR = 3.275, 95%CI = 1.673–6.409, P = 0.001; Model 2: OR = 3.111, 95%CI = 1.533–6.313, P = 0.002; Model 3: OR = 2.627, 95%CI = 1.267–5.449, P = 0.009).

In addition, low T3 syndrome was also a significant and independent predictor of the HT classification (PH or HI) after adjusting for the same potential confounders analyzed above. However, the statistical effect of low T3 syndrome on risk of HI was generally less than the risk of PH, and in Model 3, there was no significant association between low T3 syndrome and HI (OR = 1.366; 95% CI = 0.801–2.330; P = 0.253; Figure 2).

## DISCUSSION

To our knowledge, this is the first study to discuss and analyze the relationship between low T3 syndrome and HT in patients with AIS. Our results indicate that the presence of low T3 syndrome is independently associated with an increased risk for HT, particularly PH and sHT, even after adjustment for all potential and significant confounders.

In our study, nearly half the patients (49.8%) were diagnosed with low T3 syndrome after acute stroke, which is similar to results reported previously [11, 21, 22]. Also consistent with earlier reports [15, 23–26], our results indicate that compared to AIS patients without HT, those with HT tend to have a history of atrial fibrillation, greater stroke severity, and higher inflammatory indices, including leukocyte counts and fibrinogen levels. In addition, we found that high fasting blood glucose was associated with HT in AIS patients. Similarly, a prospective study of 426 patients with acute anterior stroke reported that high blood glucose and HbA1c levels were strong independent predictors of HT, and that hyperglycemia may function in the pathology of HT [27]. These results suggest those patients with low T3 syndrome who did not develop HT may have been protected by factors such as less severe stroke (lower NIHSS score), the absence of atrial fibrillation, and lower glucose levels.

We found that the type of HT exhibited by patients was significantly associated with their T3 levels. Compared to patients with HI, those with PH had significantly lower T3 levels (1.0 mmol/l vs.1.3 mmol/l, P < 0.001; Table 1) and a significantly greater fraction had low T3 syndrome (74.5% vs. 60.5%, P < 0.001; Table 1). In addition, Figure 1 shows that the more severe the radiological category of HT became, the lower the T3 levels were. This is a potentially valuable clinical finding that suggests T3 levels may not only differ between patients with and without HT, but may also be indicative of HT severity. In patients with acute stroke, it was previously shown that PH is an independent predictor of short-term adverse prognosis and poorer 90-day mortality [4]. Lee et al. suggested that PH is closely related to dysfunctional outcomes, whereas HI did not independently predict prognosis in patients with AIS [28]. Therefore, PH represents a poor clinical outcome in stroke patients [29]. Based on the adjusted multivariate regression analysis summarized in Figure 2, low T3 syndrome appears to be strongly associated with the risk of severe HT (PH), but more weakly associated with mild HT (HI). Although additional study is needed to confirm these results, it appears low T3 syndrome may be a useful predictor for severe HT in AIS patients.

The mechanism underlying the association between low T3 syndrome and the risk of hemorrhagic complications in AIS patients remains obscure. Early studies revealed that thyroid hormones cross the blood-brain barrier through active transport, and are essential for the normal brain development and function [30]. Thyroid hormones have been shown to reduce extracellular glutamate toxicity [30] and to increase the density of the brain vasculature [31] under ischemic conditions. These findings imply that thyroid hormones may be critical for reducing ischemic brain damage and provide important insight into possible biological mechanisms underlying the neuroprotective functions of T3 and hormones of the hypothalamic-pituitary-thyroid axis. In AIS patients, the occurrence of HT may result from damage to the blood brain barrier after endothelial injury [32] and secondary cerebral edema [33]. Low T3 syndrome is thought to be involved in both central and peripheral changes in the thyroid axis [34]. Of note, a recent study found that in a middle cerebral artery occlusion mouse model, increased blood-brain barrier penetration by T3 protected against ischemic damage [35]. Two other clinical studies found that lower serum free and total T3 levels were associated with ventricular system compression or brain edema in acute stroke patients [21, 36]. Brain edema and membrane disruption can exacerbate disruption of the blood brain barrier, leading to HT [32]. Notably, T3 can attenuate infarction and related edema by suppressing expression of aquaporin-4 water channels [37].

Thyroid hormones also reportedly influence the coagulation system, and coagulation/fibrinolysis disorders are common among patients with thyroid disease [38]. An early review concluded that thyroid hormone levels can significantly affect thrombocyte maturation and function [39]. Furthermore, patients with hypothyroidism showed various abnormalities of coagulation factors and fibrinolytic markers, and hypercoagulable states have been observed in cases of hyperthyroidism [39–42]. Another study showed that thyroid hormone deficiency may increase bleeding and prothrombin times and decrease the activity of some coagulation factors [43, 44]. This means that low thyroid hormone levels may shift the hemostatic system toward a state of low coagulation and high fibrinolysis, while high levels may result in a hypercoagulable and hypofibrinolytic state [43, 45, 46]. In other words, low thyroid hormone levels may increase the risk of bleeding, whereas high levels may lead to increased risk of thromboembolism [47]. Consequently, patients with hypothyroidism may be at higher risk of developing bleeding complications, including HT. We therefore suggest that maintaining normal thyroid hormone levels, especially T3 levels, may prevent the occurrence of HT in AIS patients.

### Limitations

Our study has several limitations. First, as a retrospective, single-center study, we could not establish causality. It is therefore hoped that our findings promote future, prospective, multicenter studies to further confirm our results. Second, this study needs more long-term follow-up data. Because we assessed only the early outcomes during hospitalization, the rates of HT may be underestimated, and long-term prognoses could not be evaluated. Finally, owing to limitations of the HT database, we excluded patients with thrombolytic therapy, and we did not perform a subgroup analysis of patients with and without thrombolytic therapy. We therefore need further investigation to eliminate the effect of thrombolytic therapy on the results in our study.

## CONCLUSIONS

In conclusion, our results demonstrate that low T3 syndrome may be an independent predictor of HT, especially severe HT, following AIS. We therefore suggest that monitoring T3 levels could be an effective means of preventing HT in patients with ischemic stroke.

## MATERIALS AND METHODS

### Subjects

Data were collected from the First Affiliated Hospital of Wenzhou Medical University clinical database of HT. All patients objectively diagnosed with HT consecutively between December 2013 and December 2018 were sampled in this retrospective study. This study was approved by the Institutional Review Board and Ethics Committee of the First Affiliated Hospital of Wenzhou Medical University. Because this was a retrospective study and all included data were anonymous, there was no requirement for informed consent.

The inclusion criteria for the sampled patients included: age between 18 and 90 years; (ii) suspected first-ever AIS; (iii) patients were admitted within 7 days of the stroke onset; (iv) stroke was confirmed by computerized tomography (CT) or magnetic resonance imaging (MRI) at the time of admission. Exclusion criteria included: (i) patient diagnosed with hemorrhagic stroke or transient ischemic attack (TIA); (ii) history of previous stroke; (iii) current use of thyroid drugs or previous diagnosis of thyroid disease, such as hypothyroidism, hyperthyroidism and thyroiditis; (iv) severe renal or hepatic disease; (v) patient failed to undergo a repeated CT/MRI scan; (vi) patient received intravenous thrombolytic therapy; (vii) patient died within 2 weeks after ictus; and (viii) incomplete medical records. Ultimately, 208 consecutive patients diagnosed with HT after AIS were included. Another 208 age- and sex-matched AIS inpatients without HT from the stroke center at our institution were also enrolled based on the same inclusion and exclusion criteria.

### Data collection

Patients’ baseline demographic and clinical data, including age, gender, BMI and marital status (married or single), were collected and documented. In addition, stroke risk factors were identified, including hypertension, diabetes mellitus, atrial fibrillation, dyslipidemia, previous history of stroke, current cigarette smoking, and alcohol consumption. Laboratory tests [leukocyte counts, platelets, hemoglobin, fibrinogen, glucose levels (fast blood glucose), total cholesterol and thyroid hormones] and blood pressure measurements were conducted within 24 h of hospital admission. All patients were investigated to clarify the stroke subtype according to the TOAST criteria [48]. In addition, information regarding antiplatelet, anticoagulant, and lipid-lowering treatments for acute stroke before HT were collected for all patients. The National Institutes of Health Stroke Scale (NIHSS) score was applied by well-trained neurologists within 24 h of admission and then on a daily basis. Scores on the NIHSS range from 0 to 42, and higher scores indicate more severe neurological deficits.

All patients’ thyroid hormone levels were measured at our hospital’s clinical laboratory using a completely automated AIA 600 system (Tosho Corporation). According to the manufacturer’s instructions, the reference intervals for our laboratory were as follows: T3, 1.34–2.73 nmol/L; serum free T3, 3.80-6.00 pmol/L; total thyroxine (T4), 78.38–157.40 nmol/L; serum free T4, 7.86–14.41 pmol/L; TSH, 0.34–5.60 mIU/L. Low T3 syndrome was defined as T3 levels below the lower limit of the reference interval (T3 < 1.34 nmol/L) accompanied by low or normal serum T4 and TSH levels [49–51].

### Definition and classification of HT

A brain CT scan or MRI, including diffusion weighted imaging (DWI) and T2-weighted gradient echo, was performed with all patients in this study within 24 h and 7 days (±2) after the onset of stroke. Furthermore, in order to diagnose HT promptly, an imaging examination was conducted whenever the clinical condition of a patient appeared worsen. Two experienced neurologists, blinded to the clinical data, independently examined the results of CT/MRI tests to detect the presence of HT and its subtypes.

According to the recommendations of the European Cooperative Acute Stroke Study, HT can be radiologically and symptomatically classified [52]. Through follow-up imaging assessment, HT was classified radiologically as follows. (i) Hemorrhagic infarct type 1 (HI-1): punctate petechiae along the edge of the infarction; (ii) hemorrhagic infarct type 2 (HI-2): more confluent petechiae occurring around the infarcted area, but no space-occupying effect; (iii) parenchymal hematoma type 1 (PH-1): hematoma with mild space-occupying effect (<30% infarcted area); and (iv) parenchymal hematoma type 2 (PH-2): hematoma with significant space-occupying effect (>30% infarcted area) or intraparenchymal hemorrhage outside the infarcted area [2, 29, 52]. HT was then further categorized as symptomatic or asymptomatic depending on whether deterioration of neurological function was present. Symptomatic HT (sHT) were defined as HT with a NIHSS score increase of ≥ 4, representing clinical deterioration; the rest of the HT were considered asymptomatic HT (asHT) [53, 54].

### Statistical analysis

Depending on the distribution of data, continuous variables were presented as the mean ± standard deviation or median (quartiles). Student’s t-test or the Mann-Whitney test was conducted to make comparisons, as appropriate. Categorical variables were shown as frequencies and compared using the Chi-square test or Fisher’s exact test. Statistical comparisons among the different degrees of HT were evaluated using the Kruskal-Wallis test or one-way analysis of variance (ANOVA) with T3 as the variable. After adjusting for conventional confounders and main baseline variables associated with HT identified in the univariate logistic regression analysis, multivariate-adjusted binary logistic regression was conducted to determine whether the presence of low T3 syndrome might be an independent predictor of HT, HI, PH, or sHT. Two-tailed values of P < 0.05 were considered statistically significant. SAS statistical software, version 9.2 (SAS Institute Inc, Cary, NC) and R for Windows, version 3.4.1 (http://www.r-project.org/) were used for all the statistical analyses.

## Supplementary Material

Supplementary Table 1
