# Supplementary material for: Low triiodothyronine syndrome is associated with hemorrhagic transformation in patients with acute ischaemic stroke
Source: Aging (Albany NY). 2019 Aug 27;11(16):6385–97. doi: 10.18632/aging.102195 (PMC6738409; doi:10.18632/aging.102195)
Supplement: Supplementary Table 1 [file aging-11-102195-s001.pdf]

SUPPLEMENTARY TABLE

Supplementary Table 1. Comparison of the baseline characteristics in AIS patients with low T3 syndrome stratified by HT.

Variables	Non-HT (n = 68)	HT (n = 139)	P-value*
**Demographic parameters**			
Age (years)	72.1 ± 9.7	70.4 ± 10.5	0.264
Male, n (%)	49 (72.1%)	97 (69.8%)	0.736
BMI (kg/m^2)	22.8 ± 2.8	23.3 ± 3.4	0.458
Marital status			0.799
Married, n (%)	64 (94.1%)	132 (95.0%)	
**Vascular risk factors**			
History of atrial fibrillation, n (%)	9 (13.2%)	54 (38.8%)	<0.001
History of hypertension, n (%)	46 (67.6%)	96 (69.1%)	0.836
History of diabetes, n (%)	20 (29.4%)	36 (25.9%)	0.593
History of dyslipidemia, n (%)	5 (7.4%)	12 (8.6%)	0.753
Current smoking, n (%)	37 (54.4%)	64 (46.4%)	0.278
Current drinking, n (%)	35 (55.6%)	59 (42.8%)	0.092
**Biochemistry and vital signs on admission**			
Baseline SBP (mmHg)	156.3 ± 23.2	150.3 ± 23.4	0.087
Baseline DBP (mmHg)	79.3 ± 13.3	83.6 ± 14.6	0.047
Leukocyte counts (×109/L)	6.5 ± 1.8	9.0 ± 3.5	<0.001
Platelets (×109/L)	202.0 ± 48.3	201.1 ± 67.6	0.917
Hgb (g/L)	132.0 ± 15.5	137.6 ± 16.8	0.023
Fibrinogen (g/L)	3.4 ± 0.9	4.1 ± 1.4	<0.001
Glucose levels (mmol/L)	5.8 ± 1.7	6.6 ± 2.9	0.032
Total cholesterol (mmol/L)	2.5 ± 1.7	3.7 ± 1.8	<0.001
TSH (mIU/L)	1.4 ± 0.7	1.5 ± 0.9	0.405
T4 (nmol/L)	100.2 ± 19.8	106.5 ± 19.7	0.032
T3 (nmol/L)	1.1 ± 0.1	1.0 ± 0.2	<0.001
FT4 (pmol//L)	11.5 ± 2.1	12.1 ± 2.1	0.057
FT3 (pmol/L)	4.0 ± 0.5	3.9 ± 0.6	0.078
NIHSS on admission, median (IQR)	5.0 (1.8-10.0)	7.0 (3.0-11.0)	0.033
**Stroke mechanisms**			0.236
Atherosclerotic, n (%)	55 (80.9%)	103 (74.1%)	
Cardioembolic, n (%)	11 (16.2%)	35 (25.2%)	
Lacunar, n (%)	1 (1.5%)	0	
Other causes, n (%)	1 (1.5%)	1 (0.7%)	
**Initial treatment in hospital**			
Antiplatelets, n (%)	59 (86.8%)	74 (53.2%)	<0.001
Anticoagulants, n (%)	10 (14.7%)	39 (28.1%)	0.034
Lipid-lowering agents, n (%)	66 (97.1%)	121 (87.1%)	0.022

NOTE. BMI, body mass index; DBP, diastolic blood pressure; Hgb, hemoglobin; HT: hemorrhagic transformation; NIHSS, National Institutes of Health Stroke Scale; SBP, systolic blood pressure; T3, triiodothyronine; T4, total thyroxine; TSH: thyroid stimulating hormone. *Continuous variables were compared between the groups by the Student's t-test or the Mann–Whitney test. The chi-square test was used for categorical variables.
